# Circadian phase advances in children during camping life according to the natural light-dark cycle

**DOI:** 10.1186/s40101-022-00316-x

**Published:** 2022-12-16

**Authors:** Taisuke Eto, Shingo Kitamura, Kana Nishimura, Kota Takeoka, Yuki Nishimura, Sang-il Lee, Michihiro Ohashi, Akiko Shikano, Shingo Noi, Shigekazu Higuchi

**Affiliations:** 1 Graduate School of Integrated Frontier Sciences, Kyushu University, 4-9-1 Shiobaru, Minami-ku, Fukuoka, 815-8540 Japan; 2 Department of Human Science, Faculty of Design, Kyushu University, 4-9-1 Shiobaru, Minami-ku, Fukuoka, 815-8540 Japan; 3 Research Fellow of the Japan Society for the Promotion of Science, 4-9-1 Shiobaru, Minami-ku, Fukuoka, 815-8540 Japan; 4 Department of Sleep-Wake Disorders, National Institute of Mental Health, National Center of Neurology and Psychiatry, 4-1-1 Ogawa-Higashi, Kodaira, Tokyo, 187-8553 Japan; 5 Occupational Stress and Health Management Research Group, National Institute of Occupational Safety and Health, 6-21-1 Nagao, Tama-ku, Kawasaki, Kanagawa 214-8585 Japan; 6 Laboratory of Environmental Ergonomics, Faculty of Engineering, Hokkaido University, Kita 13, Nishi 8, Kita-ku, Sapporo, Hokkaido 060-8628 Japan; 7 Faculty of Sport Science, Nippon Sport Science University, 7-1-1 Fukasawa, Setagaya-ku, Tokyo, 158-8508 Japan

**Keywords:** Circadian rhythm, Melatonin, Children, Sleep, Camping, Lighting, Physiology, Photoperiods, Non-image forming effect

## Abstract

**Background:**

It is known that the circadian rhythm phase in adults can be advanced in a natural light-dark cycle without electrical lighting. However, the effect of advanced sleep-wake timing according to the natural light-dark cycle on children’s circadian phase is unclear. We investigated the effects of approximately 2 weeks of camping life with little access to artificial lighting on children’s circadian phases. We also conducted an exploratory examination on the effects of wake time according to natural sunrise time on the manner of the advance of their circadian phases.

**Methods:**

Twenty-one healthy children (mean ± SD age, 10.6 ± 1.4 years) participated in a camping program with wake time (4:00) being earlier than sunrise time (EW condition), and 21 healthy children (10.4 ± 1.1 years) participated in a camping program with wake time (5:00) being almost matched to sunrise time (SW condition). Salivary dim light melatonin onset (DLMO) before the camping program and that after approximately 2 weeks of camping were compared.

**Results:**

DLMO was advanced by approximately 2 h after the camping program compared with the circadian phase in daily life in both conditions. In addition, the advances in DLMO were significantly correlated with mid-sleep points before the camp in both conditions (EW: *r* = 0.72, *p* < 0.01, SW: *r* = 0.70, *p* < 0.01). These correlations mean that the phase advance was greater for the children with delayed sleep habits in daily life. Furthermore, in the EW condition, mean DLMO after the camp (18:09 ± 0:33 h) was earlier than natural sunset time and there was no significant decrease in interindividual variability in DLMO. On the other hand, in the SW condition, mean DLMO after the camp (18:43 ± 0:20 h) matched natural sunset time and interindividual variability in DLMO was significantly lower than that before the camp.

**Conclusions:**

Camping with advanced sleep and wake timing under natural sunlight advances children’s circadian phases. However, DLMO earlier than sunset in an early waking condition may lead to large interindividual variability in the circadian rhythm phase.

**Supplementary Information:**

The online version contains supplementary material available at 10.1186/s40101-022-00316-x.

## Background

In modern society, electric lighting allows people to live in bright environments during the day and at night. The modern light environments that are detached from a natural light-dark cycle, i.e., sunset and sunrise, disturb circadian rhythm and sleep timing [1–3]. Circadian rhythm and sleep timing are delayed by light exposure during the early biological night [4, 5], and late circadian rhythm, late sleep timing and a concomitant lack of sleep duration are associated with adverse effects such as a decline in cognitive function [6] and increased risk of mood disorder [7], diabetes [8], and obesity [9]. The use of electric lighting at night has been shown to be associated with later circadian and/or sleep timing by field surveys in rural areas [2, 10, 11] and urban areas [12, 13], and it has been shown that the internal circadian clock synchronizes with the natural light-dark cycle in camping life without electric lighting [1] in summer and winter [3].

Although these findings provide evidence that modern light environments contribute to later circadian rhythm and that adult humans have the capability for entrainment of the circadian clock to the natural light-dark cycle, the influence of living without electric lighting on children’s circadian clock is not known. Recently, even in children and adolescents, many studies have shown that insufficient sleep due to a later circadian clock caused by electric lighting at night [14, 15] could be associated with some health problems [16–19]. Furthermore, it has been reported that children’s circadian systems are highly sensitive to light at night [20–22]. The behavior of children’s circadian rhythm in the natural light-dark cycle should be clarified in order to better understand the effects of modern light environments on the circadian clock in children. In addition, it is important to consider how the sleep-wake schedules affect the photoentrainment function of the circadian clock. In a previous study in which adults’ circadian rhythms in a natural environment were investigated [1], sleep-wake schedules were selected by participants themselves, and the effects of sleep schedule on the process by which the circadian clock entrains the natural light-dark cycle are therefore unclear.

We had the opportunity to obtain data for the circadian phases in camping programs conducted in 2016 and 2017 with the cooperation of an organization that coordinates camping programs with little access to artificial lighting for children every summer. The sleep schedule had a wake time set approximately one hour before the natural sunrise time in the 2016 camping program, whereas the wake time in the 2017 camping program was set to approximately matched sunrise time. In the two camping programs with different wake times, circadian phase changes due to camping life for approximately 2 weeks were observed in an exploratory manner. Therefore, in this study, we investigated the effects of camping life with little access to artificial lighting on children’s circadian phases, and we also conducted an exploratory examination on the effects of wake time according to natural sunrise time on their circadian phases by comparisons of results obtained in 2016 and 2017.

## Methods

### Participants and experimental conditions

Twenty-one healthy children (age range, 9–13 years; mean ± SD age, 10.6 ± 1.4 years; sixteen boys and five girls) participated in a camping program from July to August in 2016, and 21 healthy children (age range, 9–14 years; mean ± SD age, 10.4 ± 1.1 years; eighteen boys and three girls) participated in a camping program from July to August in 2017. All subjects had no existing medical conditions and were not taking any routine medications. All of the participants gave written informed consent for participation in the study, which was approved by the Ethical Committee of Kyushu University. Informed consent forms for the children were completed by the parents after confirming their child’s agreement for participation. The experiments were conducted in accordance with the Declaration of Helsinki.

**Fig. 1:**
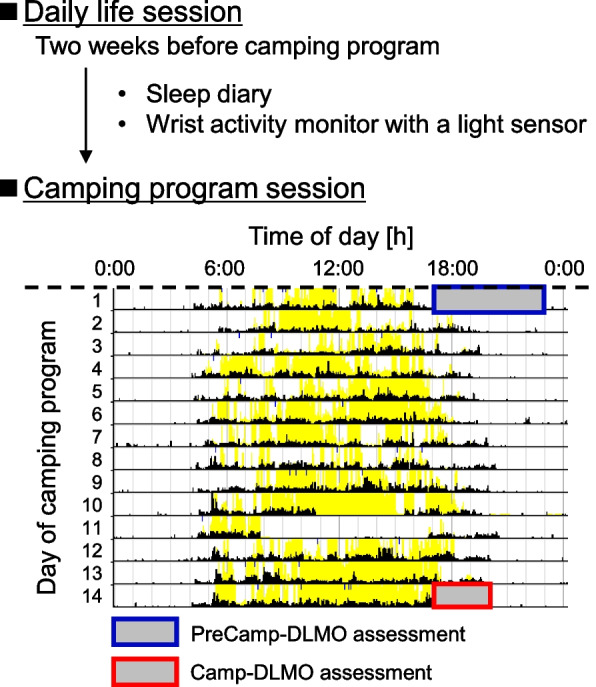
Overview of the experimental protocol with data obtained by actigraphy. Data obtained in 2017 for a participant’s activity and light exposure measured by a wrist activity monitor with a light sensor (MotionWatch 8). Black ticks denote activity and yellow ticks denote light exposure. The vertical axis shows the number of days in the camping program session and the horizontal axis shows the time of day. PreCamp-DLMO assessment was performed on the first day of the camping program both in the earlier wake time (EW) condition and the synchronized wake time (SW) condition. Camp-DLMO assessment was performed on the 11th day of the camping program in the EW condition and on the 14th day of the camping program in the SW condition

**Fig. 2:**
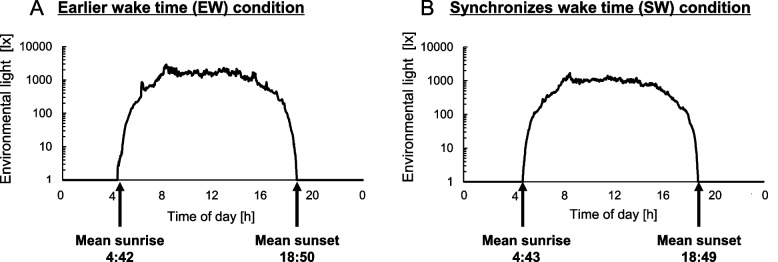
Mean environmental light illuminance in the campsite. Mean environmental light illuminance during the camping program in the campsite measured by HOBO in the earlier wake time (EW) condition (**A**) and in the synchronized wake time (SW) condition (**B**)

The camping programs were conducted by Mobilityland Corporation in Japan. The campsite was located in Tochigi Prefecture, Japan (latitude: 36.5°N, longitude: 140.2°W). According to the Sunrise Sunset Times Lookup website [23], the average times of sunset and sunrise during the camping program were 18:50 and 4:42, respectively, in 2016 and 18:49 and 4:43, respectively, in 2017. Bedtimes and wake times were set by the organizer of the camping program for 19:30 and 4:00, respectively, in 2016 (Earlier wake time condition [EW condition]: wake time being earlier than sunrise time) and for 20:00 and 5:00, respectively, in 2017 (synchronized wake time condition [SW condition]: wake time being almost matched to sunrise time). Meal times during the camping life were breakfast at 6:30, lunch at 12:00 and dinner at 17:00 in both the EW and SW conditions. At times other than sleep and meal times, the children participated in camping program events including playing in the forest, playing in a river and craftwork workshops. During the evening hours, participants prepared and ate dinner and studied in a shed with incandescent lamps. Illuminance inside the shed in the evening was less than 30 lx. For 2 weeks before the first day of the camping program, the participants were instructed to maintain their habitual bedtime and wake time and to keep a sleep diary by themselves. Sleep-wake habits and daily light exposure in that 2-week period were recorded by wrist activity monitors with a light sensor to verify compliance with instructions (in 2016: ActiSleep BT Monitor, Acti Japan; in 2017: MotionWatch 8, CamNtech). Although recording of sleep-wake habits and light exposure by the sleep diary and wrist activity monitors was also performed during the camping program (Fig. 1), the data obtained from wrist activity monitors were excluded from further analysis because the data were frequently missing because the participants sometimes forgot to wear the monitors or devices failed and because the devices used in the two conditions were different due to technical reasons. The environmental light illuminance in the campsite was recorded by light intensity loggers (HOBO CO-UA-002, Climatec). Figure 2 shows the average environmental light illuminance in the campsite measured by HOBO in the EW and SW conditions. The mean illuminances from sunrise to sunset during the camping program were 1067 ± 691 lx and 662 ± 417 lx in the EW and SW conditions, respectively.

### Circadian phase assessments

The internal circadian phase was determined by salivary melatonin secretion onset time at evening, which is known as DLMO (dim light melatonin onset) [24, 25]. The first internal circadian phase assessment for determining the circadian phase in daily life (PreCamp-DLMO) was performed on the first day of the camping program both in the EW condition and in the SW condition. The second internal circadian phase assessment for determining the circadian phase during camping life (Camp-DLMO) was performed on the 11th day of the camping program in the EW condition and on the 14th day of the camping program in the SW condition. The different assessment days between the two conditions were due to the schedule of the camping program. The circadian phase assessments were performed in a shed located at the camping site with dim lighting (< 30 lx) [25, 26] in the angle gaze when participants were seated and looking straight ahead, and saliva samples were collected to determine melatonin concentration every hour using the Salivette saliva collection device with a plain cotton plug from 17:00 until bedtime. Participants were instructed to finish dinner by 17:00 on the day of circadian assessments and sit in a chair until finishing the saliva sample collection procedure, i.e., until bedtime. Participants did not drink any water for 15 min prior to each salivary sample collection. Except for the saliva collection time, the participants were allowed to study, talk, and read. Participants were instructed to wear sunglasses if they went outside the shed to use the restroom or for other purposes during the saliva sample collection procedure. Bedtime, i.e., end time of collection of saliva samples, was defined as each participant’s habitual bedtime in the PreCamp-DLMO assessment, while it was set to 20:00 in the Camp-DLMO assessment. Salivary melatonin concentrations were quantified by using a radioimmunoassay kit (RK-DSM2, Bühlmann Laboratories). The time of DLMO, which is a marker of the circadian phase, was determined by linear interpolation between two time points at which melatonin concentration crossed the 4.0 pg/mL threshold [27, 28].

### Data and statistical analysis

Information on sleep habits (bedtime, wake time, mid-sleep point, and sleep duration) was obtained from subjective sleep diaries because actigraphy data were frequently missing because the participants sometimes forgot to wear the monitors or the devices failed, as mentioned above. Data for sleep habits were averaged data for a period of 2 weeks before the camping program and data from the day of the first circadian phase assessment to the day of the second circadian assessment as representative values of sleep habits in daily life and camping life, respectively. DLMO and sleep schedule before the camping program and those during the camping program were compared by a two-tailed paired *t* test, and DLMO, sleep-wake cycles and light exposure in the EW condition and those in the SW condition were compared by a two-tailed Welch’s *t* test.

Although all of the children who participated completed all of the procedures of the experiment, five children were excluded from further analysis because PreCamp (two children in the EW condition and one child in the SW condition) or CampDLMO (two children in the SW condition) could not be detected due to no melatonin secretion until bedtime, and six children (three children in the EW condition and three children in the SW condition) were excluded because of the omission of recording in sleep diaries. *p* < 0.05 was considered to be statistically significant in all statistical analyses. All results are presented as means ± SD.

## Results

Table 1 shows sleep-wake schedules in daily life before the camp (PreCamp) and in the camping program (Camp). The bedtime, wake time and mid-sleep point in the camping program were significantly earlier than those before the camp in both EW and SW conditions due to the forced earlier sleep-wake cycle in the camping program (EW condition: *n* = 16, all *p* < 0.0001; SW condition: *n* = 15, all *p* < 0.0001). On the other hand, there was no significant difference between sleep duration before the camp and that during the camp (EW condition: *p* = 0.12; SW condition: *p* = 0.43). A comparison of the sleep-wake cycles during the camping program between the EW and SW conditions showed that the Camp-wake time and mid-sleep point in the EW condition were significantly earlier than those in the SW condition and the Camp-sleep duration in the EW condition was significantly shorter than that in the SW condition (Table 1, EW condition: *n* = 16 and SW condition: *n* = 15, wake time: *p* < 0.001; mid-sleep point: *p* < 0.01; sleep duration: *p* < 0.05). The Camp-bedtime in the EW condition had a tendency for being earlier than that in the SW condition (*p* = 0.08).

**Table 1:** Sleep habits and circadian phase

	Earlier wake time (EW) condition (***n*** = 16)				Synchronized wake time (SW) condition (***n*** = 15)			
[h:m]	PreCamp (Mean ± SD)	Camp (Mean ± SD)	*t* value	*p* value	PreCamp (Mean ± SD)	Camp (Mean ± SD)	*t* value	*p* value
Bedtime	21:26 ± 0:54^†^	19:48 ± 0:15	7.03	< 0.0001	22:05 ± 0:52^†^	19:56 ± 0:12	10.56	< 0.0001
Wake time	6:06 ± 0:59	4:08 ± 0:17^‡^	8.92	< 0.0001	6:41 ± 0:58	4:35 ± 0:16^‡^	8.85	< 0.0001
Mid-sleep point	25:46 ± 0:51	23:58 ± 0:09^‡^	8.90	< 0.0001	26:23 ± 0:52	24:12 ± 0:13^‡^	11.21	< 0.0001
Sleep duration	8:40 ± 0:46	8:20 ± 0:25^†^	1.65	0.12	8:36 ± 0:36	8:45 ± 0:34^†^	0.80	0.43
DLMO	20:11 ± 0:49^†^	18:09 ± 0:33^‡^	9.74	< 0.0001	20:50 ± 0:45^†^	18:43 ± 0:20^‡^	10.38	< 0.0001

Each index was compared between PreCamp and Camp using the paired *t* test. Data for sleep habits (bedtime, wake time, mid-sleep point, and sleep duration) were averaged data for a period of 2 weeks before the camping program and averaged data from the day of the first circadian phase (DLMO) assessment (first day of the camping program in both conditions) to the day of the second DLMO assessment (11th day in the EW condition and 14th day in the SW condition)

*DLMO* Dim light melatonin onset

**Fig. 3:**
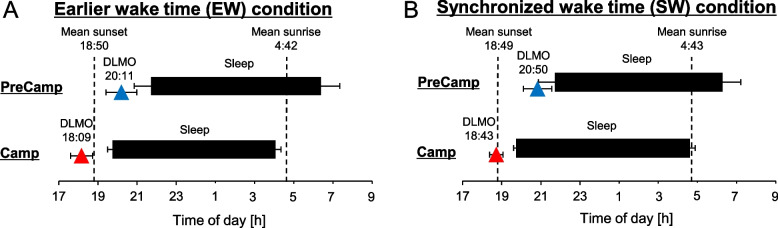
Change in mean salivary melatonin onset and mean sleep-wake timing. Mean dim light melatonin onset (DLMO) and sleep-wake timing were compared during daily life (PreCamp) and during the camping program (Camp) in the earlier wake time (EW) condition (**A**) and in the synchronized wake time (SW) condition (**B**). Salivary melatonin onset is shown as colored triangles. Blue triangles indicate PreCamp-DLMO and red triangles indicate Camp-DLMO. Bedtimes and wake times were set for 19:30 and 4:00, respectively, in the EW condition and for 20:00 and 5:00, respectively, in the SW condition

**Fig. 4:**
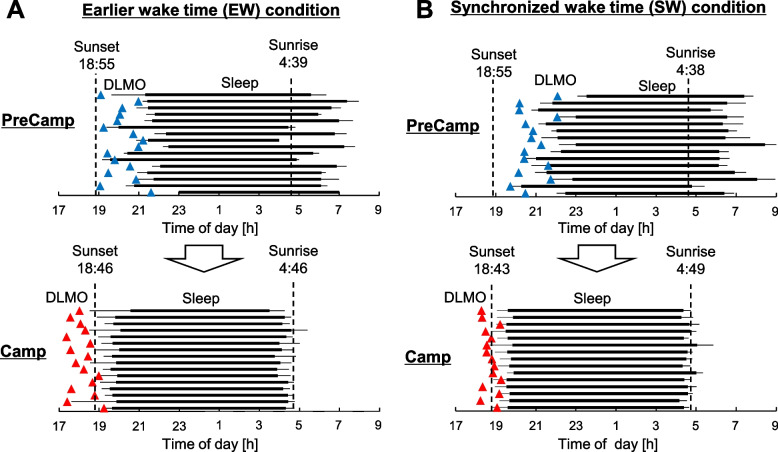
Change in individual salivary melatonin onset and individual sleep-wake timing. Individual dim light melatonin onset (DLMO) and sleep-wake timing were compared during daily life (PreCamp) and during the camping program (Camp) in the earlier wake time (EW) condition (**A**) and in the synchronized wake time (SW) condition (**B**). Salivary melatonin onset is shown as colored triangles. Blue triangles indicate PreCamp-DLMO and red triangles indicate Camp-DLMO. Bedtimes and wake times were set for 19:30 and 4:00, respectively, in the EW condition and for 20:00 and 5:00, respectively, in the SW condition

**Fig. 5:**
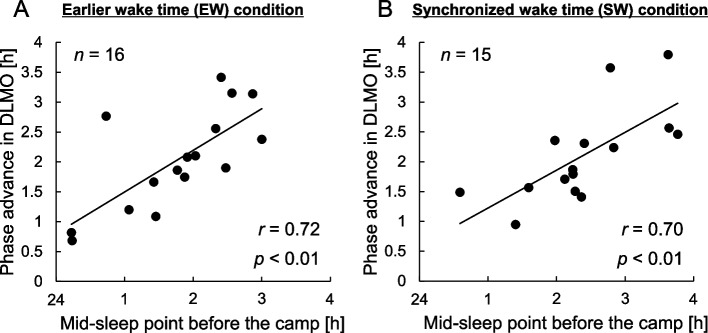
Relationships between habitual sleep timing and change in salivary melatonin onset time. Change in salivary dim light melatonin onset (DLMO), which is a marker of circadian phase, was related to habitual sleep timing (mid-sleep point) before the camping program in the earlier wake time (EW) condition (**A**) and in the synchronized wake time (SW) condition (**B**). The vertical axis shows the change in DLMO [h] and the horizontal axis shows the mid-sleep point [h] before the camping program

DLMO, which is a marker of circadian phase, at the second circadian assessment (Camp-DLMO) was significantly advanced by the camping program compared with that at the first assessment in both the EW condition and the SW condition (EW: *n* = 16, *p* < 0.0001; SW: *n* = 15, *p* < 0.0001). The average advances of DLMO were about 2 h in both conditions (EW condition: 2:02 ± 0:48 h, SW condition: 2:06 ± 0:45 h, Fig. 3). The circadian advance was significantly correlated with changes in bedtime and wake time in both conditions (EW condition: [bedtime] *r* = 0.75, *p* < 0.001, [wake time] *r* = 0.60, *p* < 0.05; SW condition: [bedtime] *r* = 0.77, *p* < 0.001, [wake time] *r* = 0.51, *p* < 0.05, Pearson’s correlation test). A comparison of DLMO between the EW and SW conditions showed that Camp-DLMO in the EW condition was significantly earlier than that in the SW condition (Table 1, *p* < 0.01). Camp-DLMO in the EW condition (18:09 ± 0:33) was significantly earlier than sunset time at the second DLMO assessment day (sunset time: 18:46, *p* < 0.01, one-sample *t* test) and the inter-individual variance of Camp-DLMO was not significantly lower than that of PreCamp-DLMO (*p* = 0.21, *F* test; Fig. 4A). On the other hand, Camp-DLMO in the SW condition (18:43 ± 0:20) almost matched sunset time at the second DLMO assessment day (sunset time: 18:43, *p* = 0.66, one-sample t-test) and the large inter-individual variance of PreCamp-DLMO was significantly decreased by the camping life (*p* < 0.01, *F* test; Fig. 4B). In addition, there were significant correlations between circadian advances and mid-sleep point before the camp in both conditions (EW condition: *r* = 0.72, *p* < 0.01; SW condition: *r* = 0.70, *p* < 0.01; Fig. 5). These results indicate that the Camp-DLMO advance was greater for children who had delayed sleep habits in daily life.

## Discussion

Children’s circadian rhythm phase, i.e., DLMO, advanced approximately 2 h after 1.5 or 2 weeks of living in accordance with a natural light-dark cycle compared with the circadian rhythm phase in daily life. We showed that the forced earlier sleep-wake cycle and light exposure by natural sunlight may have caused the children’s circadian rhythm phase to advance. We provided new evidence that children’s circadian clock may be capable of entrainment to the natural light-dark cycle.

Interestingly, mean Camp-DLMO in the EW condition was earlier than sunset time and interindividual variability in Camp-DLMO remained large and was not significantly different from PreCamp-DLMO, while mean Camp-DLMO in the SW condition almost matched sunset time and interindividual variability in Camp-DLMO was significantly lower than that in PreCamp-DLMO. Wake time that was earlier than the natural sunrise time in the EW condition may have contributed to the DLMO advance to before sunset time and to the interindividual variability in the circadian phase. The advance of DLMO to before sunset time in the EW condition would be due to the exposure of children who were already awake to natural light before sunrise (Fig. 2A). Regarding the contribution of waking up earlier than sunrise to interindividual variability in the circadian phase, it is possible that exposure around DLMO to natural light before sunset affected the results. The average environmental light illuminance around Camp-DLMO in the EW condition (18:09) was 60.7 lx (illuminance range, 10.8–161.5lx; *n* = 11 days, measured by HOBO) and the children in the EW condition were presumed to have been exposed, though not excessively, to sunlight at the start of biological night. On the other hand, the amount of light exposure around Camp-DLMO in the SW condition (18:43 ± 0:20) was 1.54 lx (illuminance range, 0.0–10.8 lx; *n* = 14 days) and was significantly less than that in the EW condition (*p* < 0.01, two-tailed Welch’s *t* test; Fig. 2). Light exposure around DLMO, i.e., the start of biological night, has been reported to contribute not only to the delay of the biological clock [4, 5, 29] but also to the expansion of individual variability in the circadian phase [30]. DLMO advanced to before sunset by waking earlier than sunrise; consequently, the environmental light before sunset was exposed to the biological night. Thus, a decrease in the inter-individual variability of the circadian phase during camping life might have been inhibited in the EW condition. Although the mechanism by which biological night light exposure contributes to increased individual differences in circadian rhythms is unknown, individual differences in light sensitivity of the circadian system may have affected the interindividual variability in the circadian phase [31, 32]. The light exposure around DLMO may seem low, but it may be sufficient to induce a non-image forming response given the high light sensitivity of children [20, 21, 33].

It should be noted that the fact that the mean Camp-DLMO in the SW condition was almost matched to sunset time may be coincidental. Therefore, a future study is needed to determine whether similar findings can be obtained under different day-length conditions, such as different seasons, different latitudes and different environmental illuminance. In addition, it is debatable whether natural light was essential to this study’s findings that the circadian phase advanced by about 2 h. To verify this, an experiment in which only the sleep schedule is advanced in an artificial lighting environment is needed as a control experiment. Furthermore, we cannot rule out the possibility that the baseline circadian phase, i.e., PreCamp-DLMO, had an effect on the earlier Camp-DLMO in the EW condition than that in the SW condition. The PreCamp-DLMO in the EW condition was significantly earlier than that in the SW condition (Table 1). However, PreCamp-DLMO did not significantly correlate with Camp-DLMO in both the EW condition (*r* = 0.32, *p* = 0.24) and the SW condition (*r* = 0.14, *p* = 0.62). This means that earlier Camp-DLMO in the EW condition cannot be explained by only early PreCamp-DLMO. Therefore, we suspect that Camp-DLMO in the EW condition was earlier than that in the SW condition due to the influence of waking before natural sunrise time rather than earlier PreCamp-DLMO.

This study has some limitations. First, the intervals between the first and second circadian phase assessments were different in the EW condition (11 days) and SW condition (14 days). The DLMO earlier than sunset time in the EW condition might be considered to be the result of detecting the state of the middle of the synchronization process due to the short interval between the first and second circadian phase assessments. However, it has been shown that the circadian rhythm entrained the natural light-dark cycle within 1 week in a previous study in which the effects of the natural light-dark cycle on adults’ circadian clock were investigated [1]. Therefore, the intervals of circadian phase assessments in both conditions are considered to be long enough to detect circadian rhythms in the synchronization state, and the differences in the manner of advance between the EW and SW conditions could be due to differences in the wake time during the camping life. Second, natural light exposure might have been different in the EW and SW conditions because weather conditions could not be controlled across conditions. In fact, the average environmental light illuminance in the campsite measured by HOBO was lower in the SW condition than EW condition (Fig. 2), suggesting that the camp program in the SW condition may have had poor weather overall (see Figure S1 for daily environmental light illuminance during the camping program). Therefore, we cannot rule out the possibility that the differences in weather conditions between the EW and SW conditions influenced the results for circadian phase. Third, since the sleep schedule was decided by the organizer of the camping program, we also could not control the sleep opportunity. One of the goals of the camping program was to improve children’s sleep habits. Therefore, not only delaying the early wake time but also extending the sleep duration was conducted in the 2017 camping program (SW condition), based on the results of the 2016 camping program (EW condition). The sleep duration was significantly different between the EW and SW conditions (show Table 1) and this difference may have affected the circadian phase results. However, the difference in sleep duration between the two conditions would have occurred due to the earlier wake time in the EW condition rather than bedtime because there was a significant difference in wake time but no significant difference in bedtime between the two conditions (show Table 1). Therefore, we assume that the earlier DLMO in the EW condition may have been influenced by earlier waking time rather than sleep duration. Finally, although the illuminance in the dim lighting condition (< 30 lx) during the saliva sampling was determined according to previous reports [25, 26], it may have been a relatively high level. Illuminance in DLMO measurements has recently been recommended to be less than 10 lx [34]. The illuminance level in the shed could not be set to a sufficiently low level because the children needed some brightness to cook dinner and study in the camping program. Although a previous study showed that melatonin is suppressed even at an illuminance of 15 lx in pre- to mid-pubertal adolescents [35], it is possible that the subjects in the previous study had stayed in a near-dark environment (~ 0.1 lx) just prior to light exposure and thus had increased light sensitivity in the circadian system [36–38]. On the other hand, the light sensitivity in the circadian system of participants in this study might be decreased in the evening because they were exposed to high-illuminance sunlight in the daytime during the camping program. In addition, it has been shown that low-color temperature lighting has a small effect on melatonin secretion even with 40 lx illuminance [39], and the dim light environment with an incandescent lamp in this study also might have had a only small effect on melatonin secretion. Furthermore, the children in this study were studying and reading during the DLMO assessment and their gaze was directed downward. Therefore, the actual cornea illuminance may have been less than 30 lx. Nonetheless, the fact that DLMO was measured at a relatively high illuminance is a limitation of this study. The DLMO in this study may not be accurate; however, it is certain that the circadian phase was advanced by the camping program since PreCamp-DLMO and Camp-DLMO were measured under the same illuminance conditions.

We showed that constructed light environments in modern society lead to a delay in children’s circadian phase or sleep-wake cycle and that the start of biological night in children, i.e., DLMO, is significantly advanced by 1.5 weeks or 2 weeks of camping life with a sleep-wake rhythm according to the natural light-dark cycle (Fig. 3). The circadian phase advance was greater for children who have eveningness sleep habits in their daily life (Fig. 5). In addition, the difference in wake times in the camping program schedules in 2016 (EW condition) and 2017 (SW condition) indicated that wake time that is earlier than natural sunrise time may lead to advanced DLMO before sunset and large interindividual variability in DLMO (Fig. 4). The finding of this study will contribute to an understanding of the mechanisms by which delayed circadian and sleep-wake rhythms develop in children in modern society [40, 41] and to the establishment of treatment for them [42]. For example, exposing a patient with a delayed circadian phase to a light environment and sleep-wake cycle in accordance with the natural light-dark cycle may result in proper timing of the circadian clock. Recently, it has become possible to artificially create skylights indoors [43], and the feasibility of treatments using natural light is increasing. Although it has been shown that morning light exposure combined with a forced advanced sleep schedule can advance the circadian phase in young adults [44] and adolescents [45], premature sleep-wake cycles may lead to circadian phase instability. Since exposure to sunlight, which is a strong zeitgeber, and a properly set sleep-wake schedule can adjust the internal biological clock to the desired timing regardless of age, i.e., adults [1] or children, and regardless of daily sleep habits, i.e., morningness or eveningness, they would contribute to reduction of negative effects due to circadian misalignments.

## Conclusions

The results of our study in which advances in circadian phases in children during camping life suggested that children’s circadian clock may be capable of entrainment to the natural light-dark cycle. Our results also showed that even when children spend time in a natural environment with sunlight, which is a strong zeitgeber, inappropriate sleep-wake schedules, e.g., waking earlier than sunrise, may lead to large interindividual variability of the circadian rhythm phase. Our findings are expected to contribute to an understanding of children’s circadian clock and to the establishment of appropriate light environments for improving health problems caused by circadian misalignment.

## Supplementary Information

**Additional file 1: Figure S1.** Daily environmental light illuminance during the camping program.

## Data Availability

The datasets analyzed in this study are not publicly available due to a privacy policy but are available from the corresponding author on reasonable requests.
