# Supplementary figures and images for: Circadian phase advances in children during camping life according to the natural light-dark cycle
Source: J Physiol Anthropol. 2022 Dec 16;41:42. doi: 10.1186/s40101-022-00316-x (PMC9756595; doi:10.1186/s40101-022-00316-x)

## Slide 1
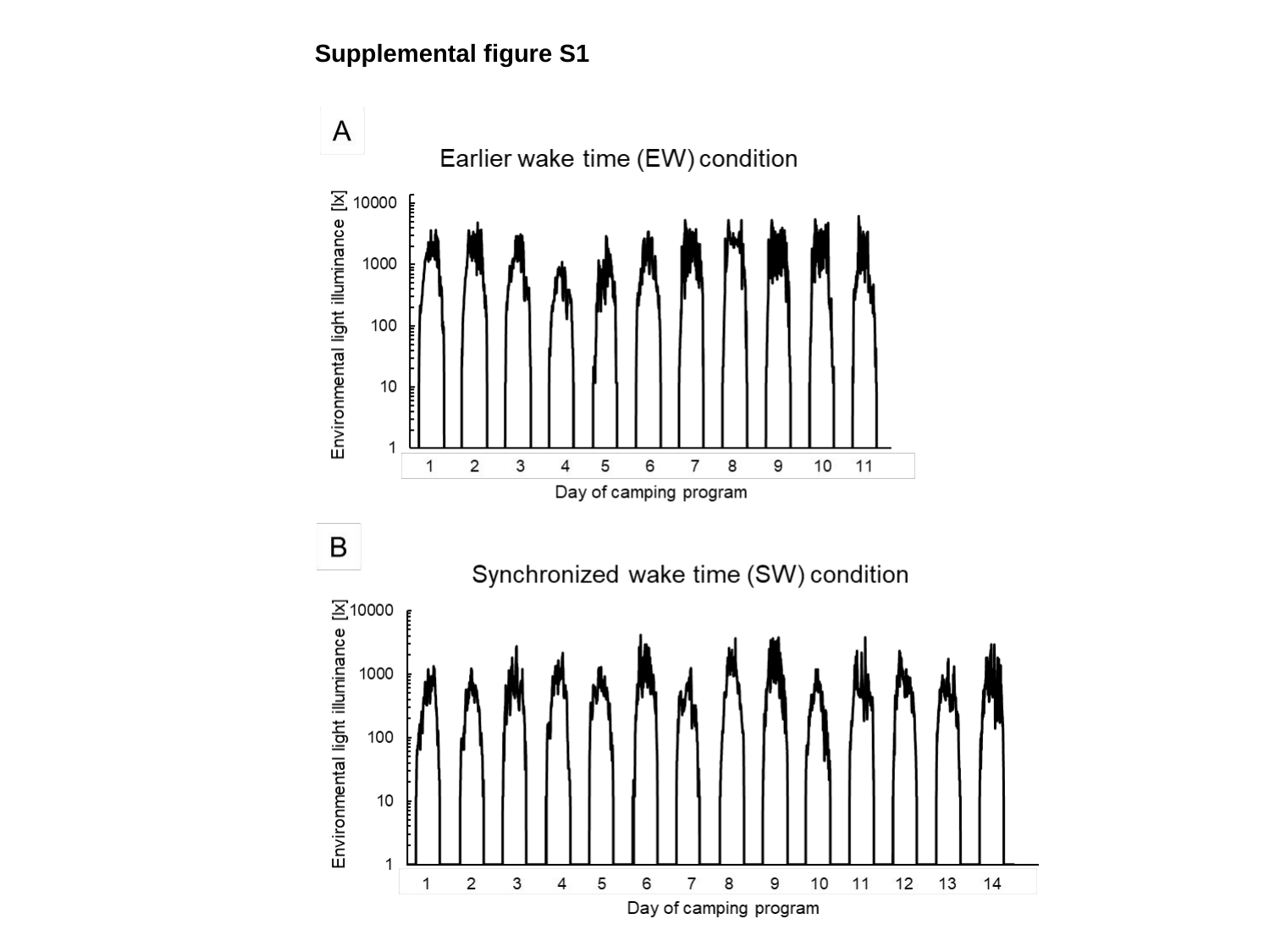

Supplemental figure S1

Supplement: Supplementary file 1 — Additional file 1: Figure S1. Daily environmental light illuminance during the camping program. [file 40101_2022_316_MOESM1_ESM.pptx]
